# Reasoning in the valuation of health‐related quality of life: A qualitative content analysis of deliberations in a pilot study

**DOI:** 10.1111/hex.13011

**Published:** 2019-12-23

**Authors:** Fabia Gansen, Julian Klinger

**Affiliations:** ^1^ Department of Health Care Management Institute of Public Health and Nursing Research, Health Sciences University of Bremen Bremen Germany

**Keywords:** deliberation, health state valuation, health‐related quality of life, MACBETH, multi‐criteria decision analysis, public value, qualitative content analysis, SF‐6D

## Abstract

**Background:**

Group deliberation can be a pathway to understanding reasons behind judgement decisions. This pilot study implemented a deliberative process to elicit public values about health‐related quality of life. In this study, participants deliberated scales and weights for a German adaption of the Short‐Form Six‐Dimension (SF‐6D) Version 2 from a public perspective.

**Objective:**

This article examines the reasons participants stated for health state valuations and investigates the feasibility of eliciting public reasons for judgement decisions in a deliberative setting.

**Methods:**

The 1‐day deliberation was guided by MACBETH as a method of multi‐criteria decision analysis and involved qualitative comparisons of SF‐6D health states and dimensions. Participants deliberated in parallel small groups and a subsequent plenary assembly. A qualitative content analysis was conducted to assess the value judgements and reasons behind them.

**Results:**

A total of 34 students participated in the study. Common reasoning was the level of impairment, marginal benefit, possibility of adjustment and expectation satisfaction. While the small groups agreed on scales for the SF‐6D dimensions, the plenary assembly did not reach consensus on one scale and dimension weights. When dimensions were prioritized, these were pain and mental health.

**Conclusions:**

While no consented value set was derived, this pilot study presents a promising approach for eliciting public reasoning behind judgements on health state values. Furthermore, it demonstrates that participants consider diverse motives when valuing health‐related quality of life.

**Abbreviations**
HRQOL: health‐related quality of life
MACBETH: measuring attractiveness by a categorical based evaluation technique
MCDA: multi‐criteria decision analysis
QCA: qualitative content analysis
SF‐6D: short‐form six‐dimension

## 1 INTRODUCTION

Preference elicitation to value health‐related quality of life (HRQOL) measures is generally conducted in large‐scale surveys of the population. These surveys can be administered using a variety of valuation techniques – such as time‐trade‐off, standard gamble and discrete choice experiments – and formats – from solely online‐based to face‐to‐face or group interviews with and without computer aids.^1^ Despite continuous development of these methods,^2^ traditional approaches to preference elicitation face several points of critique. In many cases, participants are given cognitively challenging tasks with vague and unfamiliar questioning^3^ and have little opportunity to reflect and make well‐considered judgement decisions.^4^ Additional concerns include the occurrence of inconsistent assessments^5^, ^6^ and the use of mean values which may not best mirror participants’ value judgments.^3^

In light of these issues, Hausman suggests that citizens should deliberate health state valuations to derive public values.^3^, ^7^ Taking a public perspective in valuation means considering how a health state limits the range of objectives which members of a society can pursue.^7^ This approach is in contrast to eliciting private values where citizens consider their individual objectives in health state valuation. Hausman argues that resource allocation in health care should be neutral to such individual objectives and instead consider the public value of health states.^3^ Following the argument for deliberative HRQOL valuation, this pilot study implements a consensus conference to value a German adaption of the Short‐Form Six‐Dimension (SF‐6D) Version 2. The aim of the study is to evaluate the feasibility of deliberation to elicit reasons for value judgments on HRQOL from a public perspective.

Based on a pilot study, this article examines the reasons participants give for their valuations of the SF‐6D in public deliberation. An exhaustive analysis of the applied methodology including details on the valuation approach MACBETH (Measuring Attractiveness by a Categorical Based Evaluation Technique) is presented elsewhere.^8^ In short, MACBETH is a method of multi‐criteria decision analysis (MCDA) which uses only qualitative judgements to derive numerical valuations.^9^ While MACBETH has been used in various settings within and beyond health care,^10^, ^11^ MCDA techniques have also been applied successfully in the valuation of HRQOL.^12^, ^13^ For the purpose of this study, participants used the MACBETH procedure to elicit scores and weights for the SF‐6D dimensions. Discussing the qualitative ratings needed for MACBETH was the basis of both deriving values and participants’ reasoning behind their judgement decisions when assessing HRQOL.

This research builds upon studies investigating what participants think when performing valuation tasks for HRQOL. Such studies have applied qualitative approaches such as the think‐aloud protocol for various HRQOL measures.^14^, ^15^ These analyses, however, focus on data on the individual level of study participants. With the application of deliberation for the valuation of HRQOL, this study goes beyond individuals’ reasons for judgement decisions. In brief, deliberation can be defined as non‐professional members of the public being educated about a certain topic to then consider it as a group and come to an agreed‐upon solution.^16^ Deliberation generally entails providing factual information, gathering a representative group of participants and encouraging open and reflective discussions.^17^, ^18^

In this article, we argue that deliberative settings are suited to elicit reasoning behind judgement decisions when valuing HRQOL from a public perspective. To evaluate the feasibility of eliciting public reasons on HRQOL valuation through deliberation, the analysis is performed in two steps. First, this article gives a brief overview of the valuation results and examines which reasons participants stated when expressing valuation decisions about SF‐6D health states. Second, it investigates whether the deliberative setting in this pilot study was suited to elicit public reasons for judgement decisions on HRQOL valuation. The reasons and their perspective are then discussed in the context of the pilot character of this study.

## 2 MATERIALS AND METHODS

### 2.1 Study design

**Figure 1:**
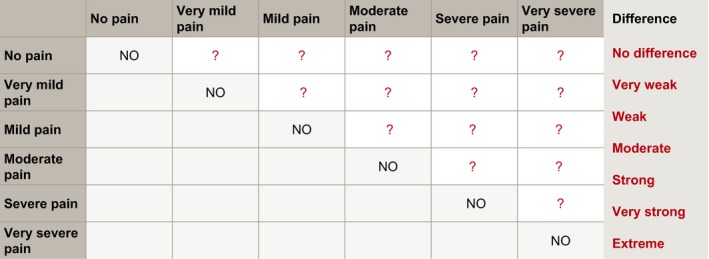
Example matrix for the scoring procedure of the dimension *pain* as presented to the participants. Note: All fields with question marks refer to the difference in attractiveness between the row and column. These differences were assessed and the corresponding fields filled in by participants

The pilot study was conducted in a 1‐day conference and applied a German adaption of the HRQOL measure SF‐6D Version 2. The descriptive system used for valuation was derived from the items of the German SF‐12 and SF‐36 corresponding to the English SF‐6D Version 2. To value the SF‐6D, deliberation first took place in six small groups which each discussed the scale of one of the SF‐6D dimensions *physical functioning*, *role limitation*, *social functioning*, *pain*, *mental health* and *vitality*. In this so‐called scoring procedure, participants gave qualitative ratings which were used to derive a quantitative scale from 0 to 1 for each dimension. To do so, each group deliberated the difference in attractiveness of two health states at a time. These states differed in the level of the group's dimension. This difference was rated on the qualitative MACBETH scale ranging from ‘no difference’ to ‘extreme difference’. An example for a judgement matrix completed by the group discussing *pain* is shown in Figure 1. The deliberative groups filled in the question marks with the differences listed in the column on the right. Participants also received detailed supporting information illustrated in Appendix S1. The small groups’ results were then brought into a plenary assembly for validation.

After eliciting a scale for each of the SF‐6D dimensions, the weighting procedure was designed to assign a weight to each dimension. In the plenary assembly, participants were first asked to rate the difference in attractiveness of a change from the worst to the best performance level one dimension at a time. In a second step, participants were to compare this change between two dimensions on the qualitative MACBETH scale. For all evaluations, participants were asked to deliberate not their personal preferences but with regard to the effects on a self‐determined and independent life. This approach was chosen to invoke reasoning from a public perspective following the public value concept suggested by Hausman.^3^

After the conference, participants evaluated the conference in a debriefing questionnaire including open questions. Details on the pilot study's evaluation and its methodology are beyond the scope of this paper and can be found in Gansen et al.^8^ In addition to the questionnaire given to the study participants, interviews with the facilitators of the deliberations as well as the software operators were conducted after the conference. In these semi‐structured interviews, the interviewees were asked to summarize the reasons stated during deliberations and elaborate how they perceived the deliberation.

### 2.2 Analysis

The numerical results of participants’ valuations were elicited with the software M‐MACBETH Version 2.5.0. This software implements the MACBETH procedure and derives numerical scales and weights from the qualitative judgements given. To assess the reasoning behind participants’ decisions, a qualitative content analysis (QCA) was performed. The QCA was conducted with transcripts of the plenary sessions and the interviews with the facilitators and software operators. The transcripts were based on audio recordings of both the conference and interviews. These recordings were transcribed verbatim following a uniform standard.^19^ The participants’ comments in the debriefing questionnaire were not included in the part of the QCA relevant to this article.^8^ The QCA was implemented with the software MAXQDA 2018.

The procedure of the QCA followed the approach suggested by Schreier.^20^ It was performed in the three steps (a) development of coding frame, (b) pilot and evaluation, and (c) main analysis. In step (a), FG applied subsumption to a sample of the text material to develop the initial coding frame inductively. Subsumption assigns all relevant text segments either to an existing subcategory or generates a new one. This data‐driven approach was used for the subcategories referring to the reasons stated by the participants. For the judgement decisions about the scales and weights of the SF‐6D dimensions, the coding frame was theory‐based as defined by the SF‐6D dimensions and MACBETH scale. In step (b), the coding frame was applied to a mostly different sample of the text material in a pilot phase. Evaluation and revision of the coding frame were based on the text‐specific and overall inter‐rater reliability calculated in percentage of agreement. Step (c) was the final phase of coding and analysis in which FG and JK applied the revised coding frame. Both coders individually coded all relevant segments of the complete text material and resolved differences by agreement. One of the coders was female, and one was male. Both coders were present during the conference and conducted two of the six semi‐structured interviews. The results of the QCA are presented here for the valuation‐ and reasoning‐related categories. The findings are supported by quotes translated from German to English. Missing speech is indicated by ellipses.

## 3 RESULTS

### 3.1 Implementation

**Figure 2:**
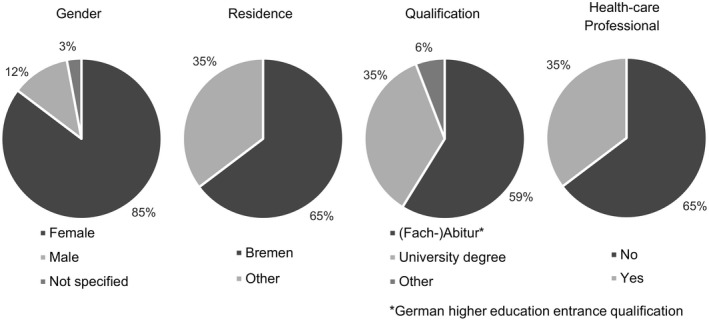
Main characteristics of pilot study participants

The conference was held on 15 December 2017 from 9.15 am to 5 pm. Thirty‐four students of the University of Bremen participated in the study. Their main characteristics are illustrated in Figure 2. All participants were students of the Bachelor's and Master's Programs of Public Health and the Master's Program Professional Public Decision Making at the University of Bremen. The small groups had 5 or 6 participants. Group assignment aimed at maximizing diversity within each group regarding gender and study programme. The pilot study was assessed by the ethics committee of the University of Bremen which waived the need for an ethics approval. The participants of the study received information material and a consent form before the conference. All participants gave informed and written consent regarding their participation and audio recordings during the conference. The interviews with the facilitators and software operators were conducted on 29 January 2018.

With regard to the context of the QCA, only students mainly with a background in health sciences took part in the study. While there was no direct link to university courses, most participants knew the researchers as lecturers. As such, there was a pre‐existing relationship between the researchers and participants of the study. To limit bias and promote open discussions, researcher engagement was restricted. Besides an introduction held by the senior researcher and assistance when questions arose, the researchers present had no active role in the conference deliberations. The deliberative sessions were facilitated by trained students of the Master's Program Professional Public Decision Making at the University of Bremen.

The entire coding frame of the QCA is made up of 9 main categories and 69 subcategories. Appendix S2 shows the complete coding frame and number of codings in each category. The overall percentage of agreement between the two coders over all categories was 76.9% after the final coding. Differences in coding were discussed and resolved through consensus. The following analysis takes only those categories of the coding frame into consideration that are relevant to the study objective. As the reasons behind valuation decisions are connected to the valuations themselves, the next section first gives a brief overview of the valuation results. It is based on the M‐MACBETH results and the QCA categories ‘Differences in attractiveness’, ‘Dimensions which should have greater weight’ and ‘Dimensions which should have smaller weight’. For the reasoning behind the participants’ value judgements, the main category ‘Reasons for evaluation of difference in attractiveness’ is assessed.

### 3.2 Valuation

#### 3.2.1 Dimension scales

The scales for each of the SF‐6D dimensions which were derived by the small groups with the M‐MACBETH software are shown in Table 1. In the plenary assembly, these results were introduced and discussed. The plenary assembly agreed on all dimension scales except for that of the dimension *pain*.

**Table 1:** Numerical scores and weights derived for the adapted German SF‐6D Version 2

	Physical functioning	Role limitation	Social functioning	Pain	Mental health	Vitality
Scoring results						
Level 1	1.00	1.00	1.00	1.00	1.00	1.00
Level 2	0.91	0.90	0.85	0.85	0.73	0.85
Level 3	0.64	0.66	0.54	0.77	0.45	0.60
Level 4	0.36	0.14	0.31	0.54	0.18	0.10
Level 5	0.00	0.00	0.00	0.15	0.00	0.00
Level 6	‐	‐	‐	0.00	‐	‐
Weighting results						
Different weights	16.13%	16.13%	16.13%	19.35%	19.35%	12.91%
Equal weights	16.67%	16.67%	16.67%	16.67%	16.67%	16.67%
No weights	‐	‐	‐	‐	‐	‐

Note

Level 1 is the best level for all dimensions. For *physical functioning*, *role limitation*, *social functioning*, *mental health* and *vitality*, level 5 is the worst level. In the dimension *pain*, level 6 is the worst level. The scale for the dimension *pain* and the weighting results were not consented by the plenary assembly.

Regarding the difference in attractiveness which participants assigned to the level dimensions, weak differences were common between the worst two levels of each dimension. Weak and very weak differences were also identified between the highest levels 1 and 2:

In the comparison of “social contacts are rarely limited” and “never limited” we saw a weak difference in attractiveness. Facilitator of group ‘Social Functioning’ in scoring session on social functioning

Individual groups chose moderate differences in attractiveness as a compromise when some participants argued for strong and others for weak differences. The assignment ‘extreme’ was predominantly given to differences in attractiveness across more than one level. In the dimension *social functioning*, for example, the deliberative group identified an extreme difference between ‘limited all of the time’ and ‘limited a little of the time’ or ‘none of the time’, respectively.

#### 3.2.2 Dimension weights

In the weighting procedure performed in the plenary assembly, different positions emerged and no consensus was reached on the dimension weights. The non‐consented results derived with M‐MACBETH are included in Table 1. Several participants were concerned about making trade‐offs between the SF‐6D dimensions. While some of these participants argued in favour of giving the same weight to all dimensions, others were not willing to make decisions on dimension weights. One participant expressed his indignation by comparing the weighting task to an impossible decision:

Yes, I absolutely agree. I mean it's not for nothing that you say choose between pest and cholera. So for me, this is exactly the same as this phrase which exists because you should not discriminate. Participant in weighting session

Those participants open to considering different weights saw a greater difference in a change from the lowest to the highest performance level for *pain* and *mental health* compared with other dimensions. Regarding the identified difference in attractiveness, the improvement in the dimensions *pain* and *mental health* was predominantly classified as ‘very strong’ or ‘extreme’. Individual participants were also in favour of a smaller difference in attractiveness for the dimension *vitality*. In her argumentation, one participant explained her choice by classifying the most severe limitations of another person:

So I thought that maybe pain could be most important. But I'm actually thinking about myself or I'm thinking if a person close to me had all of that, what would I want to free him from the most. First, I always think about pain and then I see everything on the same level and vitality a little less important. Participant in weighting session

### 3.3 Reasoning

#### 3.3.1 Level of impairment and autonomy

One of the most common arguments in participants’ judgement decisions was the level of impairment in the health state in question. This basis of reasoning was summarized in the predominant themes regarding impairment, autonomy and self‐determination.

In part, the reasons stated appeared to be specific to the dimension under consideration. For example, for *social functioning*, participants referred to the level of limitation as stated in the descriptive system of this dimension. For *role limitation*, it was argued that the effect depended on the individual's expectations and what he or she was trying to accomplish. To some extent, participants were also strongly guided by the questions posed. In many cases, participants justified their decision by stating that the health state in question had a smaller or greater effect on an independent and self‐determined life:

It is still possible to lead a self‐determined life with “mild” or “very mild pain”. Facilitator of group ‘Pain’ in scoring session on pain

#### 3.3.2 Adjustment and marginal benefit

Other recurring themes during deliberations were the prospect of tolerating or adapting to the health state in question. According to some participants, differences were less severe if you could adjust for example daily routines to health constraints. Participants also argued that in certain cases, it was possible to compensate or adapt to a limitation. For *social functioning* and the difference between ‘limited a little of the time’ and ‘some of the time’, one participant explained that for ‘limited some of the time’:

We assumed that it can't be compensated that easily and that the person may suffer if it happens regularly. That's why the difference is bigger there. Participant in scoring session on social functioning

Another approach for argumentation was the marginal benefit that could be derived from a health state in comparison with another. One day more or less in a certain state could either mean a significant improvement or rarely a difference to the affected person. An illustrative example for both points of view on marginal benefit was brought for the dimension *mental health*. On the one hand, one participant argued that every day you are not depressed is a significant gain and can be compared to Sundays that no one wants to give up. On the other hand, a different participant explained:

There, I get back to the point that I think at some point it doesn't matter if you have this one day where you are fine because one day more or less doesn't really matter since you have so many other days where you can't live a self‐determined life. Participant in scoring session on mental health

#### 3.3.3 Expectations and satisfaction

The fulfilment of expectations set both by the individuals living in a health state and those set by society was another approach for explanations. This line of argument included reasoning that certain health constraints – such as having a headache from time to time – were common and socially acceptable. Therefore, these states had little effect on a self‐determined and independent life and were classified as less severe. Participants agreed that the assessment of the impact on a self‐determined and independent life can depend on both self‐expectations and expectations of society. However, in some arguments, it was unclear which of these perspectives participants were referring to. To ensure consistent coding, ambiguous statements such as the following were coded in the subcategory ‘Self‐expectation’:

On the other side this could be about the meaning of life. If you have the feeling that you're not living up to expectations this could also be problematic. But this would also reflect on mental health. Participant in weighting session

Other themes that were identified in the participants’ arguments were how satisfied individuals are despite their health constraints. One participant argued that always being in severe pain overshadows everything and no longer allows joy of life. Another theme was arguing that some dimensions and their assessment were connected to the fulfilment of basic needs:

We also thought that this fatigue is somehow part of the basic functioning of the body. So is it able to do anything that day in the first place? Participant in scoring session on vitality

#### 3.3.4 Use of examples and other reasons

Participants also used examples as a basis for their judgement decisions. One example for this was including additional daily activities in the assessment of the dimension *role limitation*. The participants named activities such as running errands and going to the bakery as an addition to the activities listed in the descriptive system of the SF‐6D. Finally, miscellaneous subcategories which occurred less often were identified in the participants’ evaluations. These included reasoning based on intuition and other, infrequent reasons such as suffering. In some cases, participants also used unclear reasons stating ‘other’ effects or saying that certain restraints do not carry weight in comparison. As an example for quoting intuition as a basis, one participant said:

In any case, I would subjectively and intuitively say that qualitatively, there is a very big difference between going from “very little” to “no pain” and going from “very severe pain” to “severe” or vice versa, namely that the gap between “severe” and “very severe pain” should be greater. Participant in scoring session on pain

## 4 DISCUSSION

To discuss the feasibility of eliciting public reasons for valuation decisions on health states using deliberation, the following section focuses on three key issues: the main themes on reasoning identified in the deliberations, the perspective of these reasons and the suitability of a deliberative setting to elicit public reasons. A detailed discussion of the methodology applied in the pilot study is presented in Gansen et al.^8^

### 4.1 Reasons behind valuations

The results of this pilot study demonstrate that participants engaged in discussion and based their judgement decisions on various lines of argument. With regard to the valuation results on the dimension scales, the findings indicate that differences in the scales of the SF‐6D dimensions exist. In general, large differences in attractiveness were seen across more than one level of a dimension. Small differences were common between the two highest and two lowest levels of dimensions. Regarding the SF‐6D weights, one group of participants refused to derive weights and another preferred equal weights. A third group prioritized *mental health* and *pain* which corresponds to the results of the original UK valuation studies of the SF‐6D. These studies concluded that *pain*, followed by *mental health*, appeared to be the most important dimensions in determining health state values.^21^, ^22^

While the reasoning behind judgement decisions varied, recurring themes were the level of impairment and autonomy, ability to adjust, marginal benefit and expectation fulfilment. Overall, the list of themes identified through the QCA illustrates that participants of the pilot study were in fact able to express reasons for their decisions. The recap of the small group facilitators to open deliberations in the plenary assembly and in the subsequent interviews demonstrates that the arguments of the participants could be recognized and summarized. The variety of themes identified is in line with earlier qualitative work on the factors that influence the valuation HRQOL. Studies have shown that participants of valuation tasks consider tolerability and burdening family members,^23^ social circumstances and effects on family and friends,^24^ and ability to achieve goals and anticipated adaptation.^25^ Taken together, preceding research has identified several non‐health consequences connected to personal and social circumstances that influence participants’ health state valuations.^14^, ^15^ Some of these rationales, such as enjoyment, can be recognized in the reasons stated in this pilot study. Other non‐health consequences, such as ability to take care of oneself or the effect on activities or relationships, form part of the SF‐6D descriptive system. This circumstance could explain why some reasons are specific to the dimensions valued. It could also offer an explanation as to why the impact on others was not identified as a separate theme in this study. When comparing our findings to earlier qualitative research, it is important to note that other studies were based on different HRQOL measures. Moreover, participants of the comparative studies were asked for their personal preferences and not – as implemented in this study – for assessments from a public perspective.

### 4.2 Perspective of reasoning

Despite the fact that it was generally possible to identify underlying themes, not all justifications could be conclusively elicited. On the one hand, not all participants who based their judgement decisions on the same argument came to the same conclusion about the difference in attractiveness of health states. On the other hand, various decisions were not explained or made based on intuition or ‘gut feeling’. Also, the examples participants used did not clearly reveal the underlying rationale as they could not be assigned to one of the other reasoning subcategories. While intuition and ‘gut feeling’ could be seen as reasoning in themselves, some decisions were made without clearly articulating motives.

This absence of justifications could have several explanations. First, it could be difficult for participants to express their underlying values and beliefs about health in a deliberative setting. As health is a very personal topic, participants may be hesitant to share their thoughts. Hesitation could also be grounded in the fear to be judged by other participants. According to Karimi et al,^15^ valuing health is also a complex task where participants consider the practical implications of an abstract state and how it refers to their circumstances, make estimates of the consequences and weigh up these consequences. Despite the opportunity to openly reflect in the deliberative setting, this may have overwhelmed participants. Second, participants could actually share a common ground and not have the need for further explanations. Their ‘gut feeling’ about the health state in question could be the same. Third, participants may have had difficulties in taking a public perspective and may have struggled with finding ‘public’ reasons for their decisions.

Whether or not participants actually argued from a public perspective is an important point to consider in the assessment of the pilot study. Overall, most lines of argument can be classified as being based on public reasons. This is demonstrated in the use of the unspecified rationale referring to the effect on self‐determined and independent life. In that regard, an intuitive valuation on how to value a health state from the perspective of every citizen leading a self‐determined and independent life may be considered as a ‘pre‐stage’ of a public reason. From a theoretical perspective, however, one may ask whether such an intuitive valuation would be comprehensible by another free and equal citizen. If a difference in such intuitive valuations could be resolved in a deliberative exchange, we have reason to believe so.

The challenge of differentiating between public and private reasons was amplified by participants adding the phrase ‘for me personally’ when explaining their decisions. On the one hand, this phrase may simply indicate an expression of caution when entering arguments in a group discussion. On the other hand, it may also hint at the deeper problem of a possible interconnectedness of private and public reasons. This is illustrated in the line of thought from above in which a participant thinks about which limitation she would want to free a person close to her from. She starts with imagining herself in an example situation, immediately correcting herself to further assume a person close to her. This in itself would be comprehensible and relatable by other participants. Nevertheless, it is not certain whether or not she is taking a public perspective. It stands to reason that to do so, her argument would also have to extend to a random member of society and not only a friend of family member. In this particular case, however, one could also argue that her argument is simply based on human empathy and that she would come to the same conclusion if the person in question was not close to her but any other human being.

These are open questions that should be addressed with further research on the difference in public and private reasons in deliberative settings that task participants to argue form a public perspective.

### 4.3 Suitability of deliberation

To assess the suitability of deliberation to elicit public reasoning, it is important to evaluate whether the deliberative setting fulfills the requirements of deliberations. Taken as whole, the pilot study implemented the intended aspects of deliberation. This excludes the aspect of a representative group of participants as this was not a focus of the pilot study. Yet, the study did gather non‐professional members of the public who learned about a specific topic – the valuation of HRQOL – with factual information and engaged in group consideration and open, reflective discussions.^16^, ^17^, ^18^ One criterion of deliberations that was not fulfilled was the aspect of coming to an agreed‐upon solution.^16^ To ensure that non‐consented results are not due to time constraints or lacking support, longer time frames and experts in conflict resolution could facilitate future deliberations. Moreover, smaller group sizes could further encourage balanced discussions.

With regard to the suitability of deliberative settings to elicit public reasoning in the valuation of HRQOL, the pilot study provided several learnings. First, the deliberation implemented in this pilot study was a first attempt in adapting Hausman's concept to elicit public value of health states.^3^, ^7^ The deliberative setting allowed framing of the valuation tasks to a public perspective and fostered discussions about the decisions to be made. Instead of merely deciding, participants were required to express and support their valuation decisions. The facilitators and other group members also acted as a self‐control mechanism and reminded participants of explaining their arguments and taking a public perspective. The turn of perspective from private to public may provide a pathway for further research that focuses in detail on citizens’ acceptance of rationing in health care – or lack thereof. Using deliberation to elicit public reasons could contribute to a better understanding of which allocations of resources may be more acceptable than others in a publically funded health care system.

Moreover, deliberation provides a platform for participants to reflect, articulate and exchange their views on health‐related quality of life on the one hand and for researchers to elicit these views on the other. Following the findings of Karimi et al,^15^ the pilot study confirmed that valuing health is a complex task which requires time to reflect and deliberate. Beyond reflection and reasoning on an individual level, deliberation broadens the range of considerations to include those suggested by other participants. It also reveals areas in which participants lack understanding and provides the support needed to manage complex valuation tasks. As such, deliberation offers a setting to explore and address concerns that preferences on health states are not fully informed.^26^ To better inform participants about the consequences of health states, deliberations could be extended to include experts such as patient representatives and HRQOL specialists. It is important to note, however, that expert opinions could, in turn, bias participants in their judgement decisions.

### 4.4 Limitations

Overall, the findings of this study indicate that deliberation is a promising approach for eliciting reasons for decisions on health state valuation. Yet, several limitations, mainly due to the pilot character of this study, limit the transferability of its results. For this study, a convenience sample of students enrolled in Public Health and Professional Public Decision Making was chosen. Therefore, the participants had a relatively homogenous socioeconomic background. They were from one region in northern Germany, had a low average age and were mainly female. Due to their studies, at least some of the participants also had previous knowledge about HRQOL valuation or decision making. The makeup of the group may have biased this study's results and is an important point to consider in future deliberations. To include a diverse group of participants, recruiting procedures can be based on citizen's juries and use stratification.^27^ Another limitation was the use of a translated version of the SF‐6D which was not validated externally before its application. As the phrasing of the descriptive system is important to its valuation, the pilot results have to be seen against this restricting background. Notwithstanding the potential for inaccurate wording, the deliberative setting gave participants the opportunity to discuss and agree on their understanding of the descriptive system. Deliberative valuations beyond a pilot phase, however, would need to apply a validated HRQOL measure as well as consider issues such as anchoring and duration of health states. Finally, the deliberative procedure was only roughly structured and mainly constructed around the requirements of the MCDA method MACBETH. As such, the effect of deliberation cannot be separated from the methodology chosen for valuation. Future deliberative valuations should therefore attempt at standardizing the deliberative procedure and investigate the effect of public deliberation on health state values.

## 5 CONCLUSIONS

This pilot study presents a novel approach for deriving reasoning behind value judgements on HRQOL in a deliberative setting. While no consented value set was derived, participants deliberated scores and weights and justified their decisions from a public perspective. Despite the pilot character and connected limitations of this study, the results indicate that participants consider various motives when valuing health states. These influencing factors include the level of impairment and autonomy connected to health states, their marginal benefit and whether they allow expectations to be satisfied.

This study adds to findings on individual‐level motives behind health state values. To our knowledge, it is the first investigation of public reasons behind value assessments of HRQOL. As such, it can improve the understanding of health state valuations and highlight the differences between private reasoning and public reasoning. Eliciting public reasons for value judgements can also provide insights into citizens’ attitude towards resource allocation in health care. Overall, deliberative health valuation is a promising approach to examine reasoning behind HRQOL valuation. Beyond this pilot study, future deliberative valuation studies should implement a complete and standardized valuation procedure in order to investigate the effect of public deliberation on health state values.

## CONFLICT OF INTEREST

The authors declare that they have no competing interests. Julian Klinger is employed by the digital health company Newsenselab GmbH. However, his contribution to this project was largely made while working as a research assistant at the University of Bremen. Newsenselab was not involved in and did not provide funding for this study.

## Supporting information

Appendix S1 Translated guide for scoring of dimension *pain*

Appendix S2 Coding frame with number of codings in subcategories

## Data Availability

The data that support the findings of this study are available from the corresponding author upon reasonable request.
