# Supplementary material for: Reasoning in the valuation of health‐related quality of life: A qualitative content analysis of deliberations in a pilot study
Source: Health Expect. 2019 Dec 23;23(2):405–13. doi: 10.1111/hex.13011 (PMC7104633; doi:10.1111/hex.13011)
Supplement: Supplementary file 1 — Appendix S1 Translated guide for scoring of dimension pain [file HEX-23-405-s001.pdf]

Additional file 1 Translated guide for scoring of dimension *pain*

Dimension „Pain“

The dimension „Pain“ has 6 levels you are to compare:

No pain 1

Very mild pain 2

Mild pain 3

Moderate pain 4

Severe pain 5

Very severe pain 6

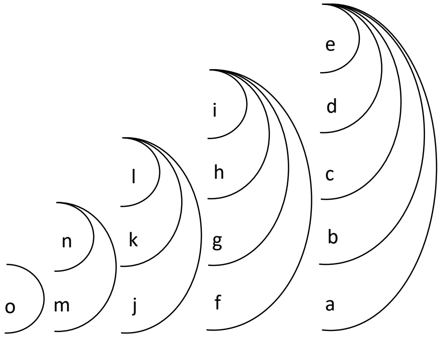

**Pain**	no	very mild	mild	moderate	severe	very severe
no	-	e)	d)	c)	b)	a)
very mild		-	i)	h)	g)	f)
mild			-	l)	k)	j)
moderate				-	n)	m)
severe					-	o)
very severe						-

The comparison is conducted with the following questions you should answer together.

How do you rate the difference in attractiveness between the two health states

a) no pain and very severe pain
b) no pain and severe pain
c) no pain and moderate pain
d) no pain and mild pain
e) no pain and very mild pain
f) very mild pain and very severe pain
g) very mild pain and severe pain
h) very mild pain and moderate pain
i) very mild pain and mild pain
j) mild pain and very severe pain
k) mild pain and severe pain
l) mild pain and moderate pain
m) moderate pain and very severe pain
n) moderate pain and severe pain
o) severe pain and very severe pain

… **with reference to a self-determined and independent life?**

There are seven possible answers. The difference can be

**extreme**	**very strong**	**strong**	**moderate**	**weak**	**very weak**	**no difference**
