# Supplementary material for: Reasoning in the valuation of health‐related quality of life: A qualitative content analysis of deliberations in a pilot study
Source: Health Expect. 2019 Dec 23;23(2):405–13. doi: 10.1111/hex.13011 (PMC7104633; doi:10.1111/hex.13011)
Supplement: Supplementary file 2 — Appendix S2 Coding frame with number of codings in subcategories [file HEX-23-405-s002.pdf]

**Additional file 2** Coding frame with number of codings in subcategories

## *Categories included in analysis*

**Difference in attractiveness**

- No difference (1)
- Very weak (4)
- Weak (4)
- Moderate (3)
- Strong (2)
- Very strong (8)
- Extreme (4)

**Dimensions which should have greater weight**

- None (8)
- Physical functioning (1)
- Role limitation (1)
- Social functioning (0)
- Pain (5)
- Mental Health (4)
- Vitality (0)

**Dimensions which should have smaller weight**

- Physical functioning (1)
- Role limitation (1)
- Social functioning (0)
- Pain (0)
- Mental Health (0)
- Vitality (3)

**Reasons for evaluation of difference in attractiveness**

- Intuition (5)
- Expectations of society (9)
- Self-expectation (10)
- Satisfaction (10)
- Tolerance or Adjustment (15)
- Marginal benefit (21)
- Level of impairment (23)
- Needs (5)
- Autonomy or self-determination (23)
- Evaluation of an example (18)
- Unclear (8)
- Other reasons (4)

## *Categories not included in analysis*

**Aspects of procedure evaluated**

- Exhaustion or excessive demand (14)
- Insecurity about procedure (9)
- Insecurity about topic (5)
- Size of plenary assembly (8)
- Time management (8)
- Organizational issues (8)
- Questioning work of small groups (13)
- Missing information (10)
- Other (6)

**Aspects of methodology evaluated**

- Understanding (15)
- Independence of dimensions (6)
- Trade-off between dimensions (9)
- Abstraction from personal level (7)
- Finding consensus (10)
- Unclear definitions (29)
- Other (4)

**Aspects of participants' behavior evaluated**

- Conversational atmosphere (17)
- Interaction (13)
- Balanced participation (3)
- Unbalanced participation (17)
- Willingness to find consensus (17)
- Unwillingness to find consensus (10)
- Moderation (6)
- Other (6)

**Aspects of research approach evaluated**

- Complexity of research question (8)
- Assumptions and implementation (6)
- Influencing results (8)
- Ethical concerns (11)
- Ethical acceptability (3)
- Practical application (6)
- Other (0)

**Suggestions for improvement**

- Information for participants (6)
- Approach for weighting (3)
- Procedure for scoring (15)
- Group organization (4)
- Time management (3)
- Other (1)
